# Specific quorum sensing-disrupting activity (A_QSI_) of thiophenones and their therapeutic potential

**DOI:** 10.1038/srep18033

**Published:** 2015-12-09

**Authors:** Qian Yang, Anne Aamdal Scheie, Tore Benneche, Tom Defoirdt

**Affiliations:** 1 Laboratory of Aquaculture & Artemia Reference Center, Ghent University, Belgium; 2 Department of Oral Microbiology, University of Oslo, Norway; 3 Department of Chemistry, University of Oslo, Norway

## Abstract

Disease caused by antibiotic resistant pathogens is becoming a serious problem, both in human and veterinary medicine. The inhibition of quorum sensing, bacterial cell-to-cell communication, is a promising alternative strategy to control disease. In this study, we determined the quorum sensing-disrupting activity of 20 thiophenones towards the quorum sensing model bacterium *V. harveyi.* In order to exclude false positives, we propose a new parameter (A_QSI_) to describe specific quorum sensing activity. A_QSI_ is defined as the ratio between inhibition of quorum sensing-regulated activity in a reporter strain and inhibition of the same activity when it is independent of quorum sensing. Calculation of A_QSI_ allowed to exclude five false positives, whereas the six most active thiophenones (TF203, TF307, TF319, TF339, TF342 and TF403) inhibited quorum sensing at 0.25 μM, with A_QSI_ higher than 10. Further, we determined the protective effect and toxicity of the thiophenones in a highly controlled gnotobiotic model system with brine shrimp larvae. There was a strong positive correlation between the specific quorum sensing-disrupting activity of the thiophenones and the protection of brine shrimp larvae against pathogenic *V. harveyi*. Four of the most active quorum sensing-disrupting thiophenones (TF 203, TF319, TF339 and TF342) were considered to be promising since they have a therapeutic potential of at least 10.

The discovery of antibiotics brought great relief from a large number of deadly illnesses in the 20^th^ century, and until now, these kind of compounds are still used as a first line therapy to treat bacterial infections in the clinic^1^,^2^. However, excessive and non-judicious use of antibiotics has resulted in the evolution of multiple drug resistant bacterial strains. Antibiotic treatments are no longer effective in some cases, and diseases caused by antibiotic resistant bacteria are currently the second leading cause of death worldwide^1^. Additionally, massive use of antibiotics in animal production also constitutes a threat to human health and to the environment^3^, and this has resulted in more strict regulations with respect to antibiotic use. One notable example is the ban on the use of antibiotics as growth promoters in animal production in Europe in 2006 (European Parliament and Council Regulation No 1831/2003). However, there are good indications that this ban could result in a higher frequency of pathogenic bacteria (such as *Salmonella* spp.), which in turn could lead to a higher frequency of infections (in animals as well as consumers). As a consequence, the development of novel strategies to control bacterial diseases, both in human and veterinary medicine will be critically important in order to ensure public health and food security in the future.

An alternative strategy to combat bacterial infections is the specific inhibition of functions required to infect the host, which has been termed antivirulence therapy^4^. This therapy consists of either inhibiting a specific virulence factor or interfering with the regulation of virulence factor expression^5^. Quorum sensing (QS) is a mechanism by which bacteria co-ordinate the expression of certain genes in response to small signal molecules. Quorum sensing has been shown to control the expression of virulence-related genes in many different pathogens, making quorum sensing disruption an interesting strategy to control bacterial disease^6^,^7^. *Vibrio harveyi* is one of the major pathogens of aquatic organisms, affecting a wide range of cultured marine animals, and causing significant losses in the aquaculture industry worldwide^8^,^9^. The species is also one of the model organisms in studies on QS in bacteria. *V. harveyi* contains a three-channel QS system, which is mediated by three types of signal molecules (HAI-1, AI-2 and CAI-1)^10^. This QS system is required for full virulence of the bacterium towards several aquatic hosts, including a highly controlled model system with gnotobiotic brine shrimp (*Artemia franciscana*) larvae^11^,^12^.

To date, several QS inhibitors have been described or claimed^6^,^7^. Brominated furanones are the most intensively studied QS inhibitors, and these compounds have been reported to disrupt QS in various Gram-negative bacteria^6^,^13^. These compounds inhibit QS in *V. harveyi* by decreasing the DNA-binding activity of the quorum sensing master regulator LuxR^14^. Unfortunately, these brominated furanones are toxic to higher organisms^15^, which means that they will not be safe for practical applications. More recently, brominated thiophenones, sulphur analogues of the brominated furanones with the same mode of action, have been synthesized, and these compounds were found to be more active than the corresponding furanones^16^,^17^,^18^. One of these compounds, (*Z*)-4-((5-(bromomethylene)-2-oxo-2,5-dihydrothiophen-3-yl)methoxy)-4-oxobutanoic acid (TF310, Fig. 1) has been reported to show the highest therapeutic index of all QS-disrupting compounds tested in our *V. harveyi* – brine shrimp model thus far, with complete protection against the pathogen at 2.5 μM and severe toxicity only being observed at 250 μM^18^. Based on these promising results, in the present study, we aimed at determining quorum sensing-disrupting activity, protective effect and toxicity of 20 thiophenones (Fig. 1). Furthermore, we propose a new parameter to describe specific quorum sensing-inhibitory activity, A_QSI_, defined as the ratio between inhibition of quorum sensing-regulated activity and inhibition of the same activity when independent of quorum sensing. Most claims with respect to quorum sensing inhibitors are based on experiments with quorum sensing signal molecule reporter strains. We recently argued that these experiments are prone to bias due to other effects compounds may have on reporter strains, and therefore, that good control experiments are required in order to exclude false positives^7^. The use of the proposed parameter A_QSI_ is a straightforward and elegant way to exclude false positives by taking into account (potential) bias related to the use of quorum sensing reporter strains.

## Results

### Impact of the thiophenones on quorum sensing-regulated bioluminescence of *V. harveyi*

In a first experiment, we determined the impact of the thiophenones on QS-controlled bioluminescence of *V. harveyi*. Wild type *V. harveyi* was grown to high cell density in order to activate QS-controlled bioluminescence, after which the thiophenones were added at 0.25, 1, 5 and 10 μM, respectively. Bioluminescence was measured 1 h after the addition of the thiophenones and our results revealed that most of the compounds were able to block bioluminescence in wild type *V. harveyi* in a concentration-dependent way. Fifteen of the 20 compounds (TF103, TF113, TF116, TF125, TF203, TF307, TF312, TF319, TF332, TF339, TF341, TF342, TF346, TF347 and TF403) were found to inhibit bioluminescence at a concentration of 0.25 μM and higher, while TF123 and TF301 significantly reduced the bioluminescence from 5 μM onwards. Additionally, TF203 could completely inhibit the QS-regulated bioluminescence at 5 μM, and TF301, TF332 and TF341 completely blocked the bioluminescence at 10 μM. Finally, TF345, TF404 and TF405 showed no effect on the bioluminescence even at the highest concentration tested (Fig. 2). The compounds had no effect on the growth of *V. harveyi* at the concentrations used (Supplementary Figure 1).

We previously argued that the identification of QS inhibitors using QS molecule reporter strains is prone to bias due to other effects compounds may have on the reporter strains and that therefore, adequate control experiments are required^19^. In order to verify that the effect observed in the bioluminescence tests with wild type *V. harveyi* was not due to toxicity, we investigated the effect of the thiophenones on bioluminescence of strain JAF548 pAK*lux*1, in which bioluminescence is independent of the QS system^18^. At 0.25 μM, none of the compounds, except for TF103, TF113 and TF116, blocked bioluminescence of JAF548 pAK*lux*1 (Fig. 3), suggesting that the luminescence inhibitory effect of the three compounds was not due to a specific inhibition of QS at this concentration. Moreover, TF123, TF125, TF301, TF312, TF332 and TF403 inhibited the bioluminescence of JAF548 pAK*lux*1 at 1 μM or higher, while TF203, TF341 and TF346 blocked the bioluminescence from 5 μM onwards. TF307, TF319, TF339, TF345, TF347, TF404 and TF405 showed no effect on the bioluminescence of JAF548 pAK*lux*1 at the highest concentration tested in this study (Fig. 3), indicating that they have low toxicity.

For the compounds that showed significant inhibition of quorum sensing-regulated bioluminescence, we further calculated the specific quorum sensing-inhibitory activity A_QSI_ as the ratio between the percentage inhibition of quorum sensing-regulated bioluminescence (in wild type *V. harveyi*) and the percentage inhibition of quorum sensing-independent bioluminescence (in JAF548 pAK*lux*1). The specific quorum sensing-inhibitory activity of compounds TF103, TF113, TF116, TF123 and TF301 was smaller than 2 at any of the concentrations tested (Table 1), and these compounds were therefore considered as false positives. Six compounds showed a high specific quorum sensing activity (>10) for at least one of the concentrations tested: TF203, TF307, TF319, TF339, TF342 and TF403.

### Impact of the thiophenones on the virulence of *V. harveyi* in the gnotobiotic brine shrimp model

Previous work in our lab showed that the virulence of *V. harveyi* in the gnotobiotic brine shrimp model system is regulated by QS^11^. Therefore, we further investigated whether the thiophenones could protect brine shrimp larvae from the pathogen in *in vivo* challenge tests. Fifteen compounds (TF113, TF125, TF203, TF307, TF312, TF319 TF332, TF339, TF341, TF342, TF346, TF347, TF403, TF404 and TF405) significantly increased the survival of brine shrimp larvae challenged to *V. harveyi* at 0.25 μM (Fig. 4). Fourteen of these significantly increased the survival of challenged brine shrimp at 1 μM (TF113 induced high mortality at this concentration), and 11 of them plus TF301 significantly increased the survival of challenged brine shrimp at 5 μM (TF332, TF341 and TF403 seemed to be toxic). At 10 μM, only 6 compounds were able to increase the survival of challenged brine shrimp larvae (TF125, TF307, TF346, TF347, TF404 and TF405), whereas for most compounds, high mortality was observed. Seven compounds offered a complete protection to the brine shrimp larvae (no significant difference in survival when compared to unchallenged larvae): TF125 (10 μM), TF203 (1 μM), TF301 (5 μM), TF307 (5 and 10 μM), TF339 (1 μM), TF341 (0.25 and 1 μM) and TF346 (5 μM).

Subsequently, in order to evaluate the relation between the inhibition of quorum sensing and the protection of brine shrimp larvae by thiophenones at different concentrations, we determined the correlation between A_QSI_ and survival of brine shrimp larvae challenged with *V. harveyi*. The correlation is significant at all concentrations tested: 0.25 μM (ρ = 0.481; *P* = 0.043), 1 μM (ρ = 0.563; *P* = 0.015), 5 μM (ρ = 0.726; *P* = 0.001) and 10 μM (ρ = 0.614; *P* = 0.007).

### Toxicity of the thiophenones to axenic brine shrimp larvae

We determined toxicity to axenic brine shrimp larvae at 0.25, 1, 5 and 10 μM. At 0.25 μM, TF103 and TF113 showed appreciable toxicity (causing >25% mortality). At 1 μM, 4 compounds induced appreciable mortality, including TF103, TF113, TF116 and TF123. At 5 μM, TF332, TF341 and TF403 also started to induce appreciable mortality, and at 10 μM, only 7 compounds showed no toxic effect (TF125, TF307, TF345, TF346, TF347, TF404 and TF405), whereas all other compounds except for TF116 and TF319 induced (almost) complete mortality (Fig. 5).

In addition, we also calculated correlations between inhibition of bioluminescence in JAF548 pAK*lux*1 and survival of axenic brine shrimp larvae treated with thiophenones. Results showed that the correlation is significant at all the concentrations we tested in this study: 0.25 μM (ρ = −0.676; *P* = 0.001), 5 μM (ρ = −0.762; *P* = 0.000), 5 μM (ρ = −0.761; *P* = 0.000) and 10 μM (ρ = −0.635; *P* = 0.002).

### Therapeutic potential of the thiophenones

In order to determine the therapeutic potential of each of the compounds, we calculated the ratio between the lowest concentration at which they increased the survival of challenged brine shrimp larvae to more than 75% and the lowest concentration at which they caused more than 25% mortality in axenic brine shrimp. Nine thiophenones showed a good therapeutic potential (ratio of at least 10): TF203, TF319, TF339, TF341, TF342, TF346, TF347, TF404 and TF405 (Table 1).

## Discussion

Due to the rise of antibiotic resistance, a significant research effort currently is devoted to the development of novel methods to control bacterial disease, and quorum sensing inhibition is one of the promising alternatives that are explored. Many compounds have been claimed to be able to inhibit quorum sensing in various pathogens^6^. Brominated furanones are one of the most intensively studied classes of quorum sensing inhibitors with a relatively well-defined mode of action^14^,^20^. However, these compounds are toxic to higher organisms, which hampers their application to control disease. Brominated thiophenones, the sulphur analogues of brominated furanones, were recently reported to be more effective and less toxic^18^. Given the promising results obtained before with a brominated thiophenones, in this study, we investigated the quorum sensing-inhibitory activity and therapeutic potential of 20 synthetic thiophenones in a highly controlled model system. Seventeen of the thiophenones included in this study were found to significantly decrease bioluminescence in wild type *V. harveyi* at one of the concentrations tested, and 12 of them decreased luminescence at the lowest concentration (0.25 μM).

One of the factors that have resulted in a boost of the quorum sensing research is the development of signal molecule reporter strains, which demonstrate a certain phenotype in response to quorum sensing molecules. An important limitation to the use of such reporter strains is that the quorum sensing-regulated phenotypes are often co-dependent on other factors and/or depend on the metabolic activity of the cells, leading to false positives^7^. In order to solve this problem, we propose the use of the specific quorum sensing-inhibitory activity A_QSI_, defined as the ratio between the inhibition of a quorum sensing-regulated phenotype (bioluminescence in this study) and the inhibition of the same phenotype in the same bacterium, but independent of quorum sensing, as a new parameter. We calculated the specific quorum sensing-inhibitory activity A_QSI_ for the thiophenones that showed significant inhibition of quorum sensing-regulated bioluminescence in wild type *V. harveyi*, and this allowed us to identify 5 compounds (TF103, TF113, TF116, TF123 and TF301) as false positives. On the other hand, six thiophenones showed a high specific quorum sensing activity (A_QSI_ > 10) for at least one of the concentrations tested: TF203, TF307, TF319, TF339, TF342 and TF403.

It has been proposed that the 5-bromomethylene side-chain of quorum sensing-inhibiting thiophenones enables them to bind to nucleophilic amino acid residues in LuxR, the quorum sensing master regulator in *V. harveyi*, thereby decreasing the ability of LuxR to activate quorum sensing target genes^18^,^21^. Most of the thiophenones that inhibited quorum sensing *V. harveyi* (with A_QSI_ > 2) also possess the bromomethylene side-chain, except for TF203, TF319 and TF342. These compouds, however contain a 5-side chain that can have the same function as the bromomethylene moiety (i.e. binding to nucleophilic amino acid residues). Some of the compounds with a 5-bromomethylene side chain (or 5-chloromethylene or 5-iodo-methylene, which have the same activity) showed no or a low specific quorum sensing inhibitory activity (i.e. TF103, TF113 and TF301). This is probably due to a low specificity of these compounds and the toxic activity that results from this (they did inhibit quorum sensing-regulated bioluminescence, but also inhibited quorum sensing-independent bioluminescence). Remarkably, the two compounds with a benzothiophenone core (TF404 and TF405) did not inhibit quorum sensing-regulated bioluminescence (despite having a bromomethylene side chain). Although the reason for this is not yet clear, we hypothesise that it might be caused by either a decreased reactivity of the bromomethylene moiety due to the presence of the benzene ring (making it less susceptible to nucleophilic attack), or steric hindrance due to the rigid benzene moiety attached to the thiophenone ring.

We further determined the therapeutic potential of the compounds in a highly controlled model system with brine shrimp larvae, and found that 9 thiophenones (TF203, TF319, TF339, TF341, TF342, TF346, TF347, TF404 and TF405) showed a good therapeutic potential. Four of these (TF203, TF319, TF339 and TF342) also showed a high specific quorum sensing-inhibitory activity. One of them (TF341) showed toxicity to *V. harveyi*, which might explain the protective effect in the brine shrimp assay. The thiophenones increased the survival of challenged brine shrimp larvae at concentrations similar to those needed to block quorum sensing-regulated bioluminescence *in vitro.* Furthermore, our results also revealed a significant positive correlation between the specific quorum sensing inhibitory activity and the protection of brine shrimp larvae, suggesting that the quorum sensing-inhibitory activity largely determines the protective effect of these compounds. Remarkably, two of the compounds with good therapeutic potential (TF404 and TF405) showed no quorum sensing-inhibitory activity at all, nor did they show any toxicity to *V. harveyi.* This suggests that these compounds interfere with virulence gene expression by interfering with a mechanism that is distinct from the three-channel quorum sensing system.

Finally, we found that there was a strong and positive correlation between toxicity of the thiophenones towards brine shrimp larvae and toxicity towards *V. harveyi* (as revealed by the inhibition of quorum sensing-independent bioluminescence), suggesting that toxicity to *V. harveyi* could be used as a good indicator for toxicity to higher organisms. Most of the thiophenones started to cause severe mortality in brine shrimp larvae at a concentration of 10 μM. However, low toxicity was observed for TF125, TF307, TF345, TF346, TF347, TF404 and TF405 at this concentration. This might be attributed to the length and position of the side-chain since it has been reported that the side-chain could significantly affect the toxicity of thiophenones by interfering their binding to essential proteins^18^,^22^. We indeed found that the compounds with the largest side chains at the 3-position of the thiophenone ring (e.g. TF339 and 341) showed the lowest toxicity. In addition to this, the two compounds with a benzothiophenone core (TF404 and TF405) also showed low toxicity.

In conclusion, in this study, we determined the quorum sensing-inhibiting activities of 20 new synthetic thiophenones towards the quorum sensing model bacterium *V. harveyi*. We proposed the new parameter A_QSI_ to determine specific quorum sensing inhibitory activity based on experiments with a quorum sensing reporter strain. We used this parameter to analyse data obtained with bioluminescence of *V. harveyi* as reporter phenotype, but it can easily be applied to any other signal molecule reporter strain. The use of A_QSI_ allowed us to exclude 5 false positives out of the 17 compounds that were able to inhibit quorum sensing-regulated bioluminescence in *V. harveyi.* We identified 6 thiophenones that were able to inhibit quorum sensing at submicromolecular levels. Further, we determined the protective effect and toxicity of the thiophenones in a highly controlled gnotobiotic model system with brine shrimp larvae. There was a strong positive correlation between the specific quorum sensing-disrupting activity of the thiophenones and the protection of brine shrimp larvae against pathogenic *V. harveyi*, and 6 quorum sensing-disrupting thiophenones were considered to be highly promising to control bacterial disease.

## Methods

### Thiophenones

**Figure 1:**
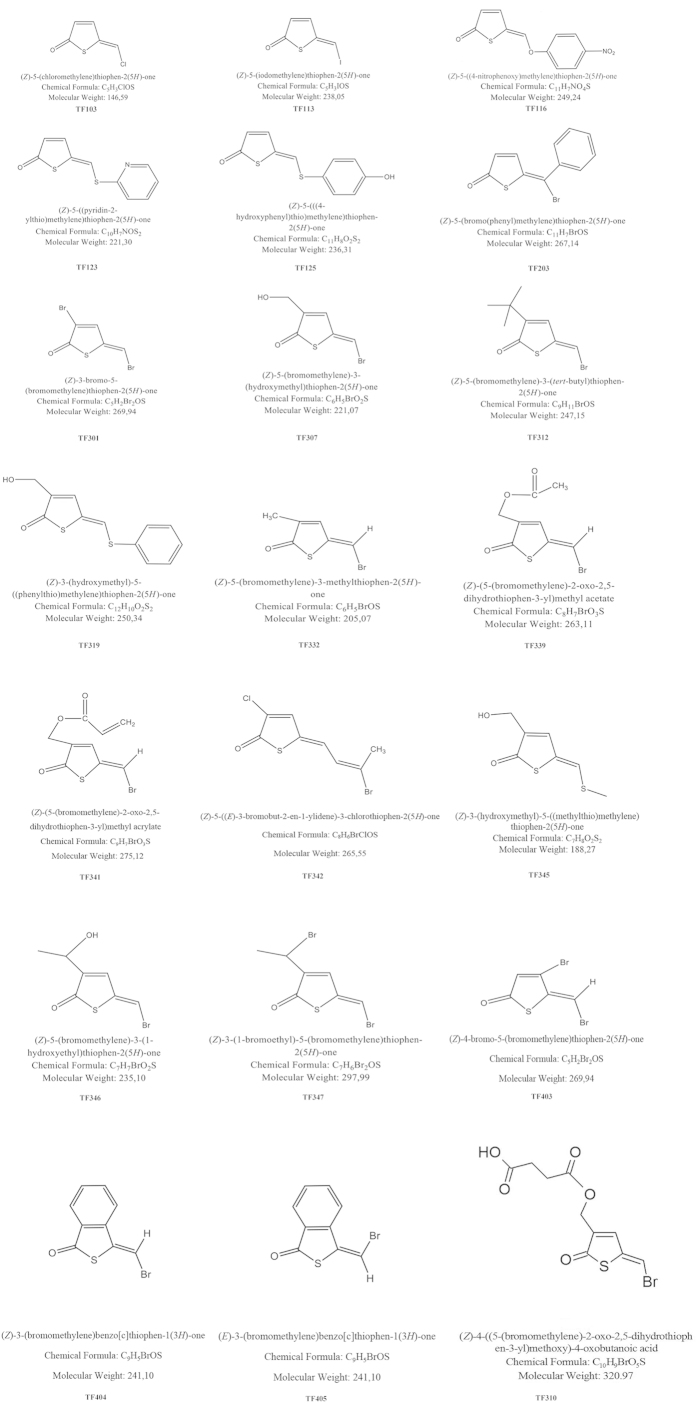
Structures of the thiophenones used in this study and compound TF310 used in a previous study^18^.

**Figure 2:**
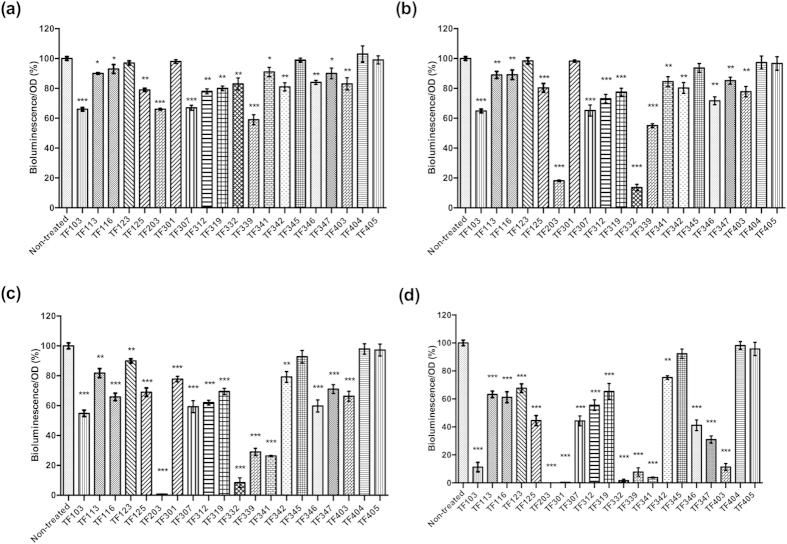
Bioluminescence of wild type *V. harveyi* in Luria-Bertani medium containing 35 g/l of sodium chloride with and without the thiophenones added at (**a**) 0.25 μM; (**b**) 1 μM; (**c**) 5 μM; (**d**) 10 μM. Luminescence measurements were performed 1 h after the addition of the thiophenones. Bioluminescence in the control treatment was set at 100% and the other treatments were normalized accordingly. The error bars represent the standard deviation of three replicates. Asterisks indicate significant differences when compared to untreated *V. harveyi* (independent samples T- test; *P < 0.05; **P < 0.01; ***P < 0.001).

**Figure 3:**
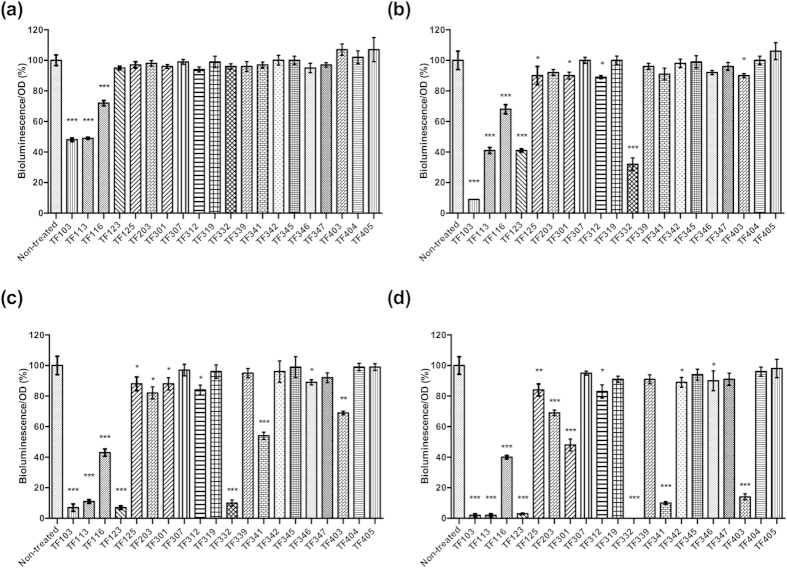
Quorum sensing-independent bioluminescence of *V. harveyi* JAF548 pAK*lux*1 in Luria-Bertani medium containing 35 g/l of sodium chloride with and without the thiophenones added at (**a**) 0.25 μM; (**b**) 1 μM; (**c**) 5 μM; (**d**) 10 μM. Luminescence measurements were performed 1 h after the addition of the thiophenones. Bioluminescence in the control treatment was set at 100% and the other treatments were normalized accordingly. The error bars represent the standard deviation of three replicates. Asterisks indicate significant differences when compared to untreated *V. harveyi* JAF548 pAK*lux*1 (independent samples T- test; *P < 0.05; **P < 0.01; ***P < 0.001).

**Figure 4:**
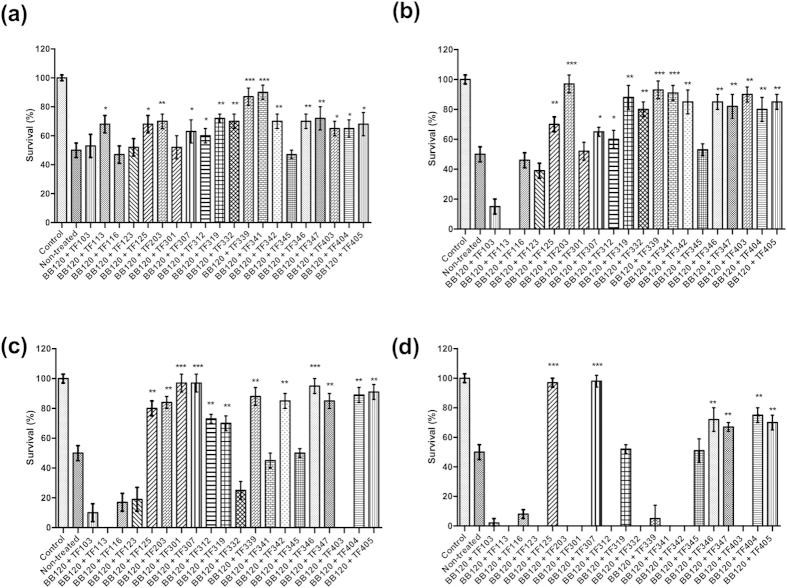
Relative percentage survival of brine shrimp larvae (average ± standard deviation of three replicates) after 2 days of challenge with wild type *V. harveyi*, without and with the thiophenones added to the rearing water at (**a**) 0.25 μM; (**b**) 1 μM; (**c**) 5 μM; (**d**) 10 μM. Survival of the unchallenged larvae was set at 100% and the other treatments were normalized accordingly. Asterisks indicate significant differences when compared to untreated brine shrimp larvae (independent samples T- test; *P < 0.05; **P < 0.01; ***P < 0.001).

**Figure 5:**
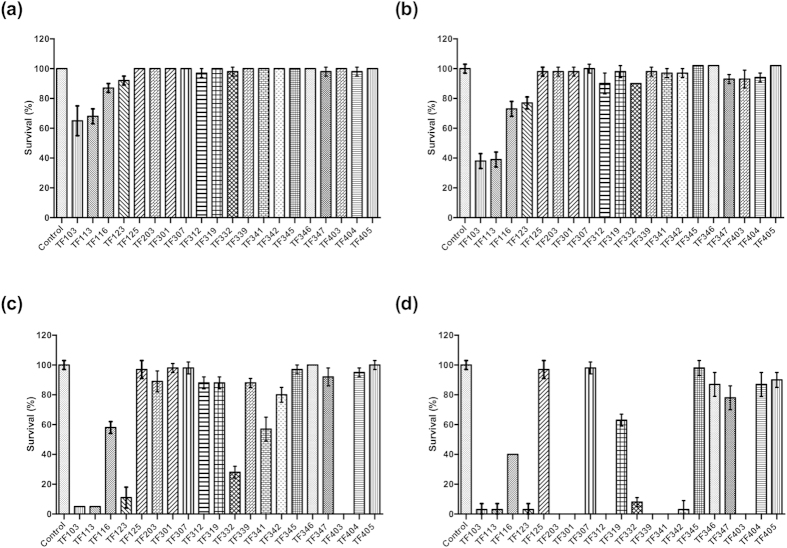
Relative percentage survival of axenic brine shrimp larvae (average ± standard deviation of three replicates) after 2 days without and with the thiophenones added to the rearing water at (**a**) 0.25 μM; (**b**) 1 μM; (**c**) 5 μM; (**d**) 10 μM. Survival in cultures without the addition of thiophenones was set at 100% and the other treatments were normalized accordingly.

The structures of the thiophenones used in this study are shown in Fig. 1. The compounds were synhtesised as described before^17^,^23^. All the thiophenones were dissolved in pure ethanol at 5 mM and stored at −20 °C.

### Bacterial strains and growth conditions

*V. harveyi* wild type strain ATCC BAA-1116 (recently reclassified as *V. campbellii*^24^; and mutant strain JAF548 pAK*lux*1^18^ were used in this study. In the latter strain, bioluminescence is independent of QS and thus, this strain is used as a control in order to verify that inhibition of luminescence in *V. harveyi* is specifically caused by QS inhibition. Both strains were cultured in Luria-Bertani medium containing 35 g/L of sodium chloride (LB_35_) at 28 °C under constant agitation (100 min^−1^). Cell densities were measured spectrophotometrically at 600 nm.

### Bioluminescence assays

*V. harveyi* wild type and mutant strain were cultured overnight and diluted to an OD600 of 0.1. The thiophenones were added at different concentrations and the cultures were further incubated at 28 °C with shaking. Then bioluminescence was measured after 1 h with a Tecan Infinite 200 microplate reader (Tecan, Mechelen, Belgium).

### Specific quorum sensing-inhibitory activity A_QSI_

The specific quorum sensing-inhibitory of the compounds at a given concentration was calculated as follows:


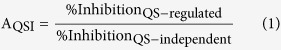


With

% Inhibition_QS−regulated_: percentage inhibition of QS-regulated bioluminescence in wild type *V. harveyi*

% Inhibition_QS−independent_: percentage inhibition of QS-independent bioluminescence of *V. harveyi* JAF548 pAKlux1

Compounds were considered as quorum sensing inhibitors if they caused a significant inhibition of quorum sensing-regulated bioluminescence and if A_QSI_ was higher than 2 at one of the concentrations tested.

### Axenic hatching of brine shrimp larvae

Two hundred milligrams of high-quality hatching cysts of *Artemia franciscana* (EG® Type; INVE Aquaculture, Baasrode, Belgium) were hydrated in 18 ml of filter-sterilized tap water for 1 h. Sterile cysts and larvae were obtained by decapsulation according to Marques *et al.* (2004). Briefly, 660 μl of NaOH (32%) and 10 ml of NaOCl (50%) were added to the hydrated cyst suspension to facilitate decapsulation. The process was stopped after 2 min by adding 14 ml of Na_2_S_2_O_3_ (10 g L^−1^). Filtered (0.22 μm) aeration was provided during the reaction. The decapsulated cysts were washed with filtered (passed through 0.22-μm membrane filter) and autoclaved (moist heat at 121 °C for 15 min) artificial seawater (containing 35 g l^−1^ of instant ocean synthetic sea salt, Aquarium Systems, Sarrebourg, France). The cysts were resuspended in a 50-ml tube containing 30 ml of filtered, autoclaved seawater and hatched for 28 h on a rotor (4 min^−1^) at 28 °C with constant illumination (c. 2000 lux). The axenity of cysts was verified by inoculating one ml of culture water into 9 ml of marine broth and incubating at 28 °C for 24 h. After 28 h of hatching, batches of 30 larvae were counted and transferred to fresh, sterile 50-ml tubes containing 30 ml of filtered and autoclaved seawater. Finally, the tubes were returned to the rotor and kept at 28 °C. All manipulations were performed in a laminar flow to maintain sterility of the cysts and larvae.

### Brine shrimp challenge tests

The impacts of the thiophenones on the virulence of *V. harveyi* were determined in a standardized challenge test with gnotobiotic brine shrimp larvae as described by Defoirdt *et al.* (2005) with some modifications. A suspension of autoclaved LVS3 bacteria^25^ in filtered and autoclaved seawater was added as feed at the start of the challenge test at 10^7^ cells ml^−1^, and *V. harveyi* was added at 10^5^ CFU ml^−1^. The thiophenones were added directly into the brine shrimp rearing water at different concentrations. Brine shrimp cultures, to which only autoclaved LVS3 bacteria were added as feed, were used as controls. The survival of the larvae was counted 48 h after the addition of the pathogens. Each treatment was carried out in triplicate and each experiment was repeated twice to verify the reproducibility. In each test, the sterility of the control treatments were checked at the end of the challenge by inoculating 1 ml of rearing water to 9 ml of marine broth and incubating the mixture for 2 days at 28 °C.

### Statistics

Data analysis was carried out using the SPSS statistical software (version 21). Unless stated otherwise, all data were analysed using independent samples *t*- tests.

## Additional Information

**How to cite this article**: Yang, Q. *et al.* Specific quorum sensing-disrupting activity (A_QSI_) of thiophenones and their therapeutic potential. *Sci. Rep.* **5**, 18033; doi: 10.1038/srep18033 (2015).

## Supplementary Material

Supplementary Information

## Figures and Tables

**Table 1:** Comparison of the specific quorum sensing inhibitory activity (A_QSI_) and the therapeutic potential of the thiophenones used in this study.

Compound	Specific quorum sensing inhibitory activity A_QSI_				Therapeutic potential		
	0.25 μM	1 μM	5 μM	10 μM	[Therapeutic]^a^ (μM)	[Toxic]^b^ (μM)	[Toxic]/[Therapeutic]
TF103	0.7	0.4	0.5	0.9	NO	0.25	−
TF113	0.2	0.2	0.2	0.4	NO	0.25	−
TF116	0.3	0.3	0.6	0.7	NO	1	−
TF123	NS	NS	0.1	0.3	NO	5	−
TF125	7.0	2.0	2.7	3.5	5	>10	>2
TF203	17.0	10.3	5.5	3.2	1	10	10
TF301	NS	NS	1.9	1.9	5	10	2
TF307	33.0	35.0	13.7	11.2	5	>10	>2
TF312	3.7	2.5	2.4	2.6	NO	10	−
TF319	20.0	23.0	7.8	3.9	1	10	10
TF332	4.3	1.3	1.0	1.0	1	5	5
TF339	10.3	11.3	23.4	10.2	0.25	10	40
TF341	3.0	1.7	1.6	1.1	0.25	5	20
TF342	19.0	10.0	5.3	2.3	1	10	10
TF345	NS	NS	NS	NS	NO	>10	−
TF346	3.2	3.5	3.7	5.9	1	>10	>10
TF347	3.3	3.8	2.9	7.8	1	>10	>10
TF403	17.0	2.2	1.1	1.0	1	5	5
TF404	NS	NS	NS	NS	1	>10	>10
TF405	NS	NS	NS	NS	1	>10	>10

NS: no significant inhibition of QS-regulated bioluminescence in *V. harveyi*.

NO: no therapeutic potential observed.

^a^The lowest concentration at which the survival of challenged brine shrimp larvae increased to more than 75%.

^b^The lowest concentration at which the compounds cause >25% mortality in axenic brine shrimp larvae.

## References

[b1] WHO. Antimicrobial Resistance: Global Report on Surveillance. World Health Organization, Geneva, Switzerland. 71pp. (2014).

[b2] Defoirdt T. Antivirulence therapy for animal production: filling an arsenal with novel weapons for sustainable disease control. PLoS Pathog. 9, e1003603 (2013). 24130477 10.1371/journal.ppat.1003603 PMC3795005

[b3] Cabello F. C. Heavy use of prophylactic antibiotics in aquaculture: a growing problem for human and animal health and for the environment. Environ. Microbiol. 8, 1137–1144 (2006). 16817922 10.1111/j.1462-2920.2006.01054.x

[b4] Clatworthy A. E., Pierson E. & Hung D. T. Targeting virulence: a new paradigm for antimicrobial therapy. Nature Chem. Biol. 3, 541–548 (2007). 17710100 10.1038/nchembio.2007.24

[b5] Defoirdt T., Sorgeloos P. & Bossier P. Alternatives to antibiotics for the control of bacterial disease in aquaculture. Curr. Opin. Microbiol. 14, 251–258 (2011). 21489864 10.1016/j.mib.2011.03.004

[b6] Kalia V. C. Quorum sensing inhibitors: An overview. Biotechnol. Adv. 31, 224–245 (2013). 23142623 10.1016/j.biotechadv.2012.10.004

[b7] Defoirdt T., Brackman G. & Coenye T. Quorum sensing inhibitors: how strong is the evidence? Trends Microbiol. 21, 619–624 (2013). 24126008 10.1016/j.tim.2013.09.006

[b8] Austin B. & Zhang X. H. *Vibrio harveyi*: a significant pathogen of marine vertebrates and invertebrates. Lett. Appl. Microbiol. 43, 119–124 (2006). 16869892 10.1111/j.1472-765X.2006.01989.x

[b9] Defoirdt T., Boon N., Sorgeloos P., Verstraete W. & Bossier P. Quorum sensing and quorum quenching in *Vibrio harveyi*: lessons learned from *in vivo* work. ISME J. 2, 19–26 (2007). 18180744 10.1038/ismej.2007.92

[b10] Ng W.-L. & Bassler B. L. Bacterial Quorum-Sensing Network Architectures. Annu. Rev. Genet. 43, 197–222 (2009). 19686078 10.1146/annurev-genet-102108-134304 PMC4313539

[b11] Defoirdt T. & Sorgeloos P. Monitoring of *Vibrio harveyi* quorum sensing activity in real time during infection of brine shrimp larvae. ISME J. 6, 2314–2319 (2012). 22673627 10.1038/ismej.2012.58 PMC3504963

[b12] Pande G. S. J., Natrah F. M. I., Sorgeloos P., Bossier P. & Defoirdt T. The *Vibrio campbellii* quorum sensing signals have a different impact on virulence of the bacterium towards different crustacean hosts. Vet. Microbiol. 167, 540–545 (2013). 24055027 10.1016/j.vetmic.2013.08.021

[b13] Manefield M. *et al.* Halogenated furanones inhibit quorum sensing through accelerated LuxR turnover. Microbiology 148, 1119–1127 (2002). 11932456 10.1099/00221287-148-4-1119

[b14] Defoirdt T. *et al.* The natural furanone (5*Z*)‐4‐bromo‐5‐(bromomethylene)‐3‐butyl‐2(5*H*)‐furanone disrupts quorum sensing‐regulated gene expression in *Vibrio harveyi* by decreasing the DNA‐binding activity of the transcriptional regulator protein LuxR. Environ. Microbiol. 9, 2486–2495 (2007). 17803774 10.1111/j.1462-2920.2007.01367.x

[b15] Natrah F. M. I. *et al.* The impact of quorum sensing on the virulence of *Aeromonas hydrophila* and *Aeromonas salmonicida* towards burbot (Lota lota L.) larvae. Vet. Microbiol. 159, 77–82 (2012). 22465799 10.1016/j.vetmic.2012.03.014

[b16] Witsø I. L. *et al.* Thiophenone and furanone in the controlof *Escherichia coli* O103:H2 virulence. Pathog. Dis. 70, 297–306 (2014). 24391047 10.1111/2049-632X.12128

[b17] Benneche T., Herstad G., Rosenberg M., Assev S. & Scheie A. A. Facile synthesis of 5-(alkylidene)thiophen-2(5*H*)-ones. A new class of antimicrobial agents. RSC Adv. 1, 323–332 (2011).

[b18] Defoirdt T. *et al.* A quorum sensing-disrupting brominated thiophenone with a promising therapeutic potential to treat luminescent vibriosis. PLoS One 7, e41788 (2012). 22848604 10.1371/journal.pone.0041788 PMC3404956

[b19] Defoirdt T., Pande G. S. J., Baruah K. & Bossier P. *Antimicrob. Agents Chemother.* **57**, 2870-2873. 10.1128/AAC.00401-13 PMC3716134 23545532

[b20] Janssens J. C. A. *et al.* Brominated furanones inhibit biofilm formation by *Salmonella enterica* serovar *Typhimurium*. Appl. Environ. Microbiol. 74, 6639–6648 (2008). 18791004 10.1128/AEM.01262-08 PMC2576703

[b21] Benneche T., Chamgordani E. J. & Scheie A. A. Reaction of (*Z*)-5-(Bromomethylene)thiophen-2(5H)-one with Some Nucleophiles in Search for New Biofilm Inhibitors. Synth. Commun. 43, 431–437 (2012).

[b22] Steenackers H. P. *et al.* Structure–activity relationship of brominated 3-alkyl-5-methylene-2(5*H*)-furanones and alkylmaleic anhydrides as inhibitors of *Salmonella* biofilm formation and quorum sensing regulated bioluminescence in *Vibrio harveyi*. Bioorg. Med. Chem. 18, 5224–5233 (2010). 20580562 10.1016/j.bmc.2010.05.055

[b23] Benneche T., Chamgordani E. J., Herstad G., Tannæs B. S. M. & Scheie A. A. 5-Alkylidenethiophen-2(5H)-ones as biofilm inhibitors. *S*ubmitted.

[b24] Lin B. *et al.* Comparative genomic analyses identify the *Vibrio harveyi* genome sequenced strains BAA-1116 and HY01 as *Vibrio campbellii*. Environ. Microbiol. Rep. 2, 81–89 (2010). 20686623 10.1111/j.1758-2229.2009.00100.x PMC2912166

[b25] Verschuere L. *et al.* Microbial Control of the Culture of *Artemia* Juveniles through Preemptive Colonization by Selected Bacterial Strains. Appl. Environ. Microbiol. 65, 2527–2533 (1999). 10347038 10.1128/aem.65.6.2527-2533.1999 PMC91373

