# Supplementary material for: Specific quorum sensing-disrupting activity (AQSI) of thiophenones and their therapeutic potential
Source: Sci Rep. 2015 Dec 9;5:18033. doi: 10.1038/srep18033 (PMC4673447; doi:10.1038/srep18033)
Supplement: Supplementary Information [file srep18033-s1.pdf]

Specific quorum sensing-disrupting activity (A_{QSI}) of thiophenones and their therapeutic potential – supplementary information

Qian Yang, Anne Aamdal-Scheie, Tore Benneche and Tom Defoirdt

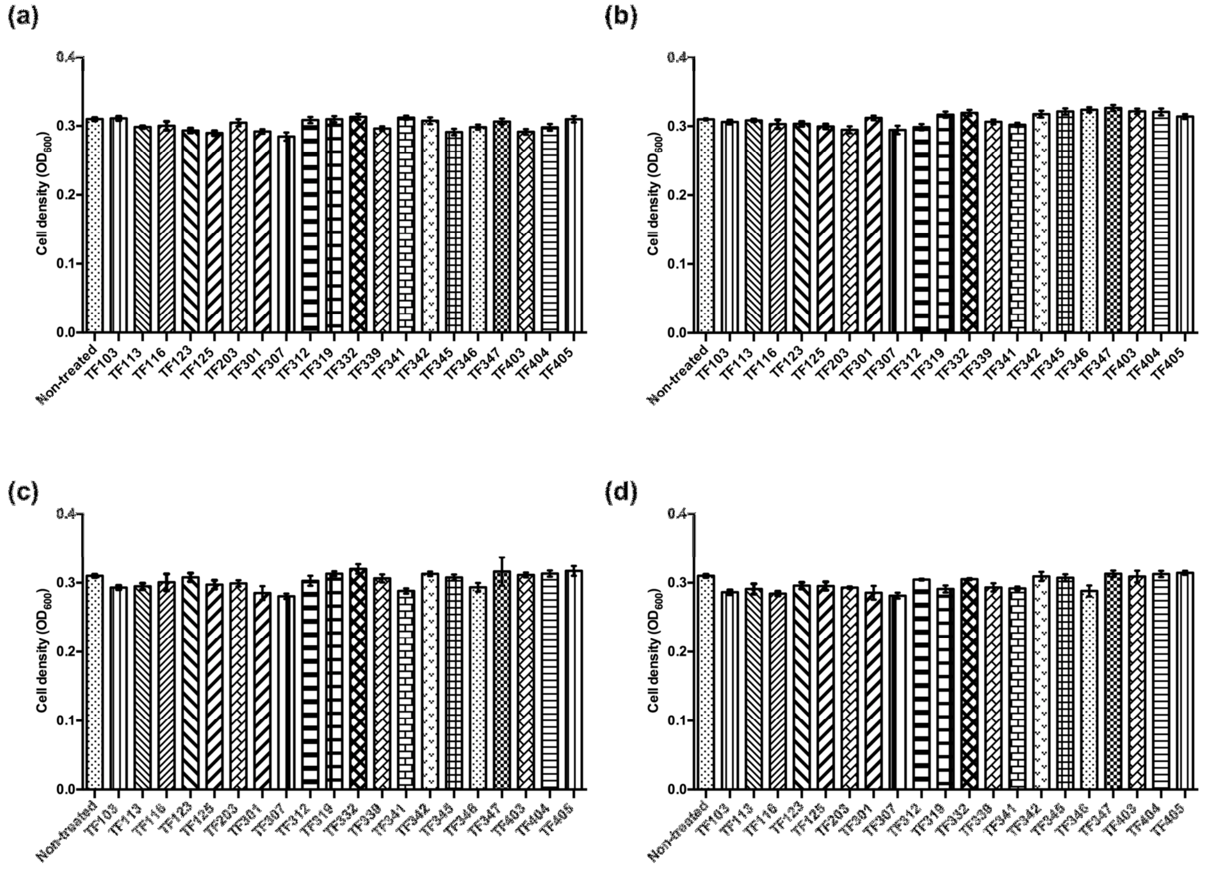

Supplementary Figure 1. Growth of wild type *V. harveyi* in Luria-Bertani medium containing 35g/l of sodium chloride with and without the thiophenones added at (a) 0.25 µM; (b) 1 µM; (c) 5 µM; (d) 10 µM. Growth measurements were performed 1h after the addition of the thiophenones. The error bars represent the standard deviation of three replicates.
